# Land-based crop phenotyping by image analysis: Accurate estimation of canopy height distributions using stereo images

**DOI:** 10.1371/journal.pone.0196671

**Published:** 2018-05-24

**Authors:** Jinhai Cai, Pankaj Kumar, Joshua Chopin, Stanley J. Miklavcic

**Affiliations:** Phenomics and Bioinformatics Research Centre, University of South Australia, Mawson Lakes, SA 5095, Australia; College of Agricultural Sciences, UNITED STATES

## Abstract

In this paper we report on an automated procedure to capture and characterize the detailed structure of a crop canopy by means of stereo imaging. We focus attention specifically on the detailed characteristic of canopy height distribution—canopy shoot area as a function of height—which can provide an elaborate picture of canopy growth and health under a given set of conditions. We apply the method to a wheat field trial involving ten Australian wheat varieties that were subjected to two different fertilizer treatments. A novel camera self-calibration approach is proposed which allows the determination of quantitative plant canopy height data (as well as other valuable phenotypic information) by stereo matching. Utilizing the canopy height distribution to provide a measure of canopy height, the results compare favourably with manual measurements of canopy height (resulting in an *R*^2^ value of 0.92), and are indeed shown to be more consistent. By comparing canopy height distributions of different varieties and different treatments, the methodology shows that different varieties subjected to the same treatment, and the same variety subjected to different treatments can respond in much more distinctive and quantifiable ways *within* their respective canopies than can be captured by a simple trait measure such as overall canopy height.

## Introduction

Plant breeders seek to identify new cereal plant varieties with potential for increased biomass, grain yield and greater resilience to adverse environmental conditions. To support plant breeder efforts and to accelerate the plant breeding process itself, new software methodologies and hardware technologies for improved genotyping and phenotyping need to be developed [1]. Given the advances made in modern genetics and genomics during the last two decades, the bottleneck would appear to lie with the crop phenotyping pipeline. One small step toward achieving the ideal pipeline involves the quantitative tracking of a multitude of phenotypic traits during a season. In the particular context of *in situ* field studies and specifically of cereal crop assessment, a number of determinant phenotypic crop traits are relevant: canopy coverage, canopy height, canopy health (NDVI), as well as the appearance of major growth stages (esp. tillering, heading and flowering times). In this paper we report on some particular phenotyping aspects of a field experiment which looked into the influence of nitrogen application on the growth behavior of ten Australian wheat cultivars. We focus attention here on the specific trait of canopy height distribution as derived from a time sequence of stereo images of field plots of the ten cultivars. This is one of two ideas we present in this paper.

Canopy height distribution, as opposed to just canopy height, has not been considered as a phenotypic trait due mostly to the difficulty in obtaining such detailed information, as well as the difficulty in its quantitative utilization [2–4]. However, the distribution of leaf height within a canopy is arguably a better quantitative measure than canopy height alone, which, incidentally, is often determined manually and therefore open to subjective error. When combined with canopy area distribution, height distribution can provide a more accurate measure of *in situ* biomass, which in turn has been shown to be strongly correlated with grain yield [5, 6]. By inference, knowledge of canopy distribution development and understanding of its relation to environmental influences during a season can guide a plant breeder’s decision of which varieties to continue assessing and which to discard.

Imaging and image analysis methods have gained popularity recently for applications in a range of plant phenotyping situations [7–14]. Although, most techniques and methods for plant phenotyping [10, 15, 16] have been designed and are presently used for assessing growth and development in controlled environments such as glasshouses, a variety of image-based, field phenotyping platforms are now being considered [4, 16–19]. However, their full utilization is hampered by the lack of a suitable framework for the analysis of the images that are being captured.

In the context of field phenotyping, the processing requirements are (a) full automation, requiring little if any user input for high throughput applications (plant breeders typically assess several thousand plots in any one season and at any one location), (b) robustness to images taken under different imaging conditions, and, (c) quantitative accuracy of extracted information. In a series of papers outlining plant image analysis methods for high throughput processing of large volume image data of plants grown in closed environments [10, 20–23], heavy reliance was made of controlled lighting and uniform background conditions to achieve these goals. The convenience of uniform conditions, however, is not replicated in the field. This poses some considerable difficulty for the expert and non-expert alike. However, for the non-expert, but likely user, the difficulties are compounded by the lack of knowledge of image processing and image analysis methods. For example, if camera settings such as focal length and camera position are varied, a re-calibration of camera images is required in order to re-estimate camera lens distortion in order to obtain accurate absolute quantitative information on plant traits that can be used for comparisons over time and between varieties. This requirement is not often recognized let alone met although it is essential for a truly quantitative analysis.

Thus, a second idea we convey in this paper is that of an innovative but simple approach to obtaining absolute quantitative measures directly from stereo image pairs as an alternative to the inconvenience of plant and crop scientists needing to become familiar with camera calibration and processing software. We have developed a software solution that will calibrate camera images without need of any additional reference measures. This allows the user to take full advantage of the vision system and deduce quantitative plant traits accurately. Embedded in the approach, is the notion of normalized disparity from stereo images. This allows the algorithm to be robust to small vibrations of the imaging system. As a result, the proposed approach is suitable for plant phenotyping in field conditions.

In summary, the main contributions of this study are (a) an automated field phenotyping framework, engineered to provide objective and accurate estimates of canopy height and canopy height distribution, and (b) a user friendly approach to robust camera calibration for quantitative field phenotyping applications.

## Experimental setup and data collection

### Field experiment and mobile imaging platform

The field trial was conducted at Mallala, South Australia (latitude = −34.457062, longitude = 138.481487), in a 5 × 12 split block design with a total of 60 plots consisting of ten spring wheat (*Triticum aestivum* L.) varieties, two fertilizer treatments and three replicates. The field was sown on July 8, 2016 at a seeding rate of 45 g/plot. To mitigate the effects of border rows, an additional plot, not included in the analysis, was planted at the beginning and end of each row of plots. Plots were 1.2 × 4 m^2^, with a gap of approximately 1 metre between columns and rows. Half of the replicates were treated with a top dressing of a standard mix of 16:8:16 N-P_2_O_5_-K_2_O at 37.5 *g*/*m*^2^ on August 12, 2016 followed on September 8, 2016 with a top dressing of Urea at 4.3 *g*/*m*^2^. The remaining 30 plots received no treatment. Imaging of the field took place between August 23, 2016 and December 2, 2016, at a desired rate of twice per week, weather permitting. A total of 23 image sets were actually captured during the season, but only a subset before the heading growth stage is included here.

**Fig 1:**
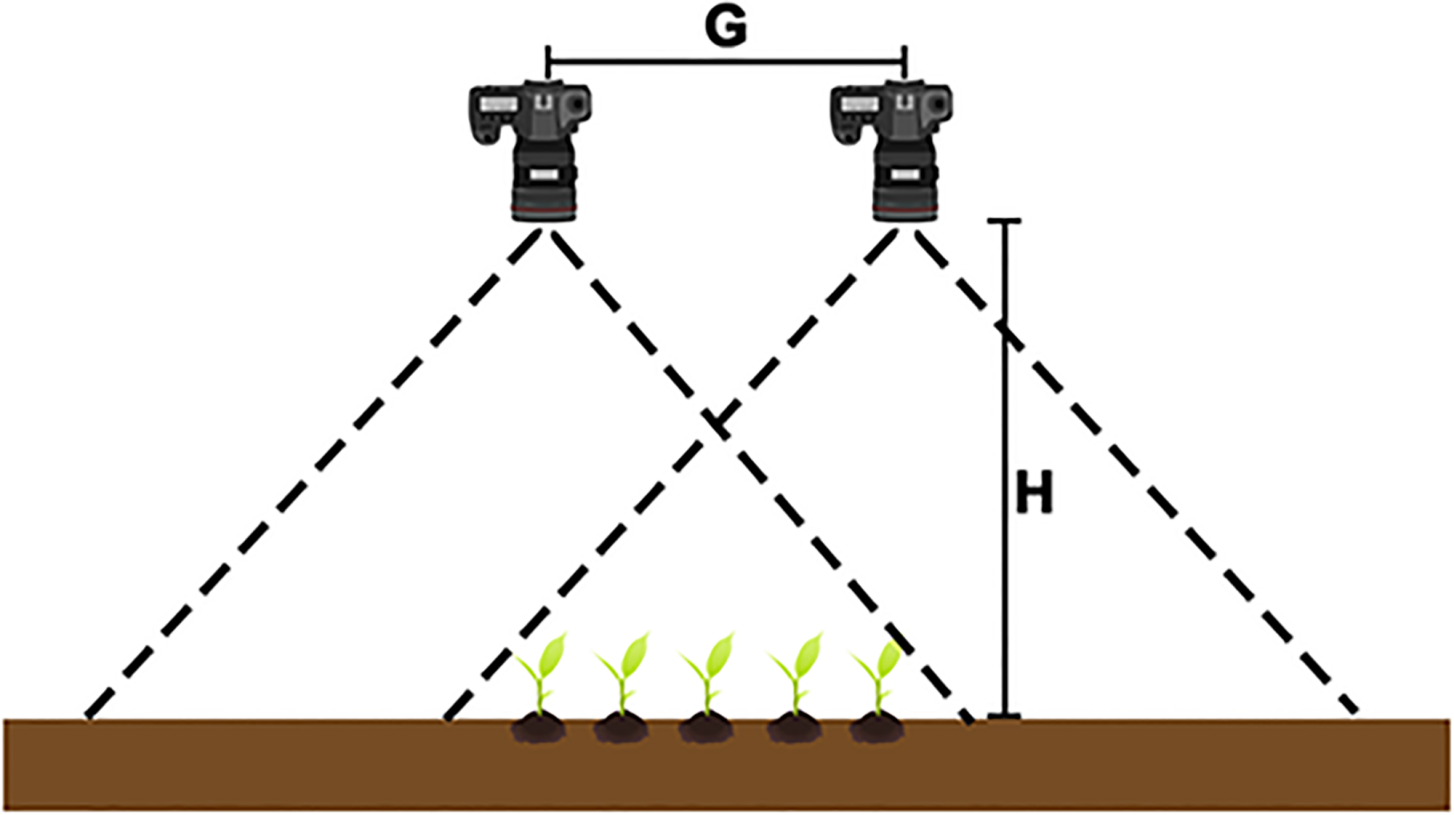
Stereo camera pair arrangement. Schematic showing the physical arrangement of the stereo pair of RGB cameras. Camera height above ground level, *H*, was fixed at 190 cm. The distance between camera apertures was fixed at G = 20 cm. The cameras used were of the make Canon EOS 60D with a focal length of 18 mm and resolution of approximately 0.04 cm per pixel.

A manually propelled wagon was used for the capture of these images. A pair of Canon EOS 60D digital cameras for stereo image capture were mounted on a central overhead rail 20 centimetres apart and 190 centimetres above ground level, on a steel frame supported by a base of four wheels. The overhead rail was also capable of supporting other imaging sensors (a third RGB camera with an oblique view plus a multispectral camera), but these do not feature in this report. A schematic of the camera arrangement is shown in Fig 1. Manual focus of the stereo camera pair was used during all imaging sessions with cameras focused at 2 metres and 1.5 metres during early and late plant growth stages, respectively. Camera settings were as follows: focal length—18 millimetres; aperture—*f*/9.0; ISO setting—automatic; and, exposure time—1/500 seconds. With such camera settings the image resolution was found to be approximately 0.04*cm* per pixel. Cameras were synchronized to capture images within 1 millisecond of each other. An X-rite colour checker was attached to the left side of the wagon, so that it would be visible from the perspective of the left-side camera.

### Manual height measurements

The height of each plot was measured manually at each imaging session using a one metre ruler with markings every centimetre. The height of a plant was defined as its highest point, including the spike but excluding the awns protruding from spikes. At earlier stages when spikes were not present the heights recorded corresponded to the heights of the uppermost level of leaves. Measurements at 3–5 positions across uniform regions of each plot were taken and averaged to give a single measure per plot and per time point. These average heights provide the most representative measure of whole plots. In cases where a few spikes or flag leaves protruded above the remainder of the canopy, these were ignored.

## Results and discussion

### Automated height estimation: Evaluation and consistency

**Fig 2:**
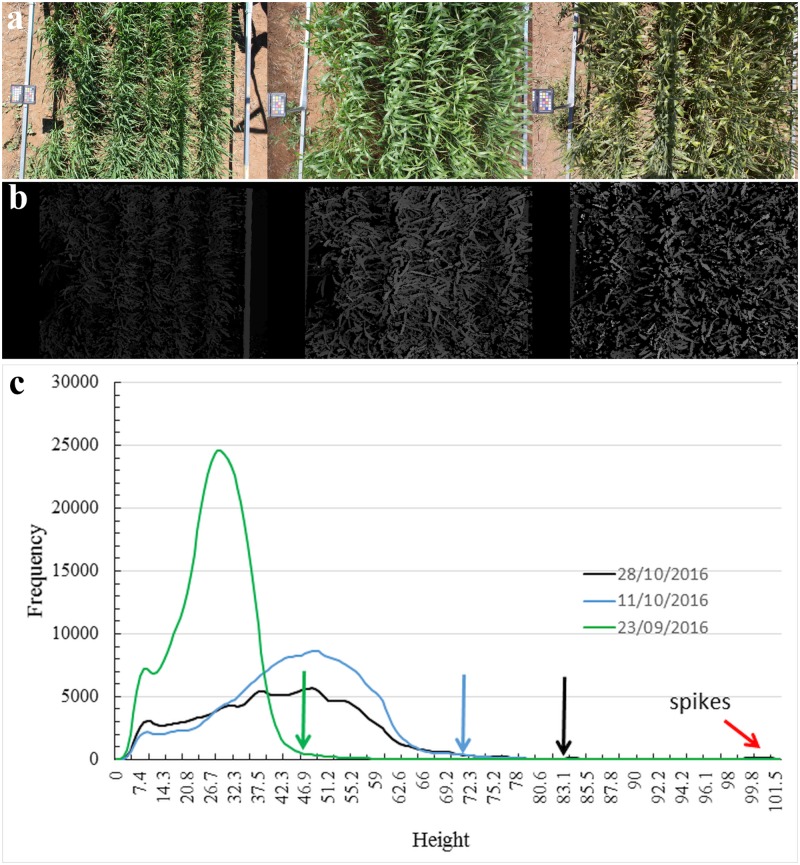
Components in the height estimation pipeline. (a) Example single camera images of a field plot (Plot 52 of 60—variety: Gregory and treatment: Fertilized), taken at three different time points (from l. to r.: 23/09/2016, 11/10/2016 and 28/10/2016). (b) Corresponding depth maps using the disparity mapping technique described in the paper. (c) Frequency histograms showing leaf pixel height distributions on these days. Note that the vertical axis gives the number of plant pixels in the image at a particular height (horizontal axis). Colour coded vertical arrows indicate the locations of the common percentage threshold (98%) used to determine canopy height. The red arrow indicates the height of the spike distribution present on day 28/10/2016. Note also that a common rectangular region was considered in obtaining the data.

From the (calibrated) stereo image pairs we applied a stereo matching procedure to generate so-called *depth maps*, defined as 3D graphs of plant height as a function of position within the respective plots. From this detailed depth map information we determine the theoretical plot heights by thresholding the top 2% of the depth map histograms as indicated in Fig 2. We compared these derived measures with the manual measurements of average plot height. Clearly, with this theoretical definition, plot height will not correspond to the highest point of the plot, but be somewhat less than this. We return to this point later in the discussion.

The estimated canopy heights of Plot 52 (variety: Gregory and treatment: Fertilized) on days 23/09/2016 and 11/10/2016, 44*cm* and 67*cm*, respectively, are seen to be very close to the manual measures of 44*cm* and 65*cm*. However, the estimated canopy height of 82.8*cm* on 28/10/2016 differed from the manual measure of 98*cm* by a non-negligible amount. This difference of 15.2*cm* is attributed to the lengths of a non-negligible number of spikes that were present at this later stage, that appeared above the average leaf canopy and that were included in the manual measurement. In the depth map distribution shown in Fig 2, we indeed find a small peak at around 100*cm* height, which is due to those same spikes. However, our threshold estimate of canopy height, which excludes the top 2% of the distribution, also excludes the contribution from these spikes.

To avoid any confusion that might arise as a result of such discrepancies, we focus attention in the remainder of this paper on canopy height estimation for the cases that precede spike appearance. However, we point out that it is possible to estimate the average height of spikes (and even their average length) by first detecting spikes in original images [24] and then deducing heights and lengths in the identified regions of the depth maps.

**Fig 3:**
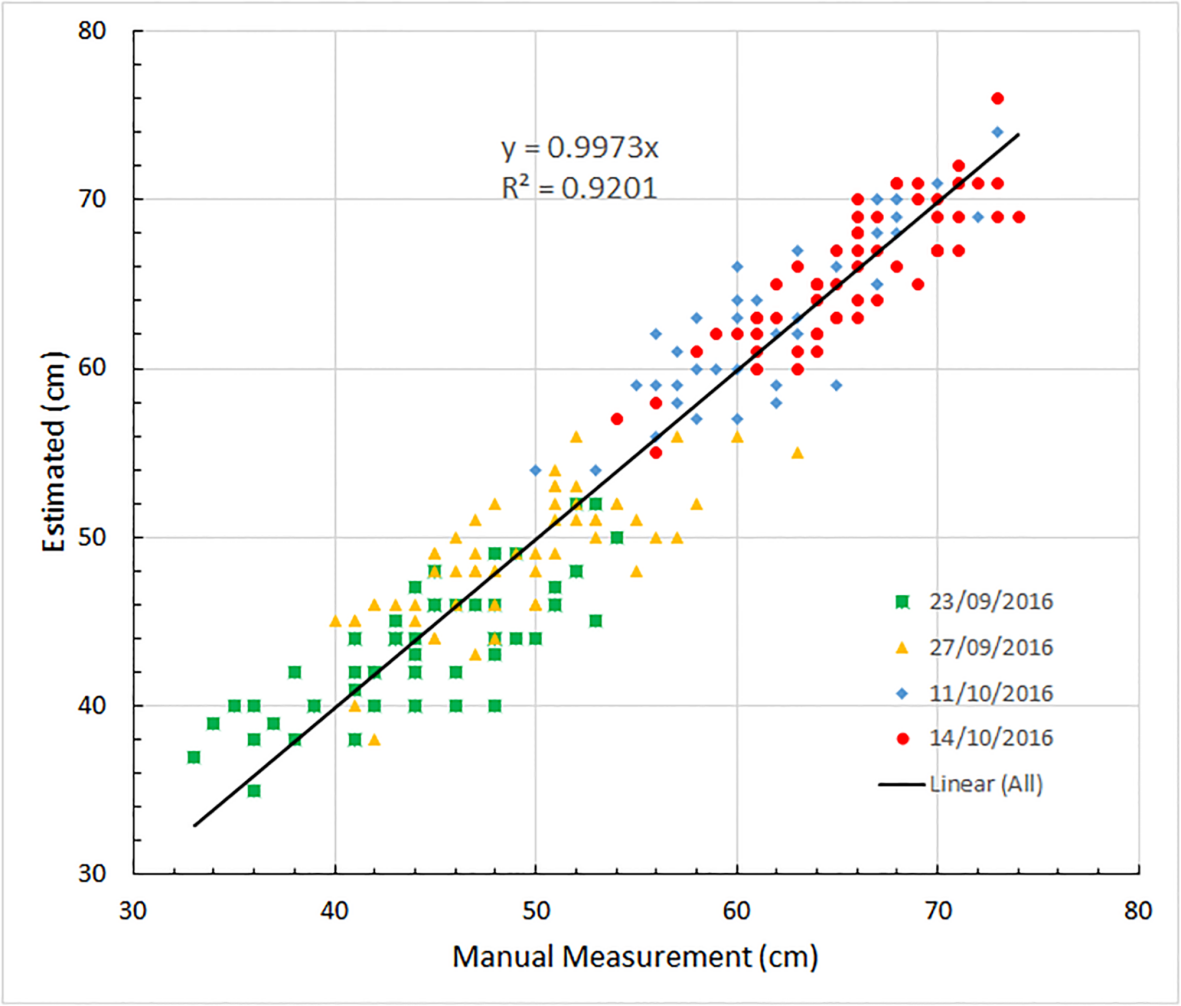
A whole-of-data comparison of automated height estimation and manual measurement. Manual measurements of canopy height for the field trial of 60 plots taken on four occasions are compared with the canopy height estimated using depth map estimation from stereo images. The solid line is the line of best fit to the data which, having a slope of 0.9973, is close to the ideal 45° line. Note that manual measurements as reported are the result of averages of several observations sampled across the plot, and therefore are prone to some degree of variation. Data points with different colors are from the four different days on 23/09/2016 (green squares), 27/09/2016 (yellow triangles), 11/10/2016 (blue diamonds) and 14/10/2016 (red circles).

We applied the stereo matching algorithm to automatically generate depth maps (*i.e*. height distribution graphs) from images taken of the 60 plots over two months during the season up to the time of spike appearance. Thresholding of the resulting histograms in the manner described above gave estimates of canopy height for the 240 data points from four days that are compared in Fig 3 with corresponding manual measurements. The comparison, represented by a straight line of best fit *y* = *αx*, where *x* and *y* denote the measured and estimated canopy heights, respectively, produced values of *α* = 0.9973, *R*^2^ = 0.9201, and a standard deviation of 2.9 cm. The close to ideal (*i.e*., unbiased) quantitative comparison between estimate and measured height, irrespective of growth stage and independent of field illumination (which did vary over the season), can be compared with the results obtained by others [2, 3, 17] who, through analogous comparisons with ground truth measurements, achieved lower *R*^2^ comparisons and greater bias |*α* − 1|≫0 values (*e.g*. *R*^2^ = 0.543 and *α* = 0.842 in the case of [25] or *R*^2^ = 0.92, *α* = 0.817 and a shift of about 10 cm in the best case [3]). We can conclude that our automated procedure is arguably more robust and comparably accurate to manual determination of height.

From the differences between estimated and manually measured canopy heights at the later stage of growth, and from their agreement at early and mid-growth stages, a number of inferences can be drawn. First, if the protocol followed for manual measurements was to exclude spikes from consideration, then good agreement between theoretical and manual measures would likely result throughout the season, even with our convenient choice of a simple threshold (top 2%). Secondly, with our depth map it is possible to identify spikes, if any are present, and moreover it is possible to quantify their particulars. This is evidenced by the presence of the small peak indicated by the red arrow in Fig 2. Finally, it is of course feasible that one could employ a more sophisticated procedure to automatically estimate canopy height that would include spikes in the estimate, if these were abundant. For example, fitting a suitable distribution model to the top 5% or top 10% of the depth map histogram, although it would add to the computational effort, could establish canopy height as the height of the abundant spikes or alternatively as height of the uppermost leaf level if spikes were either not present or present in fewer numbers.

**Fig 4:**
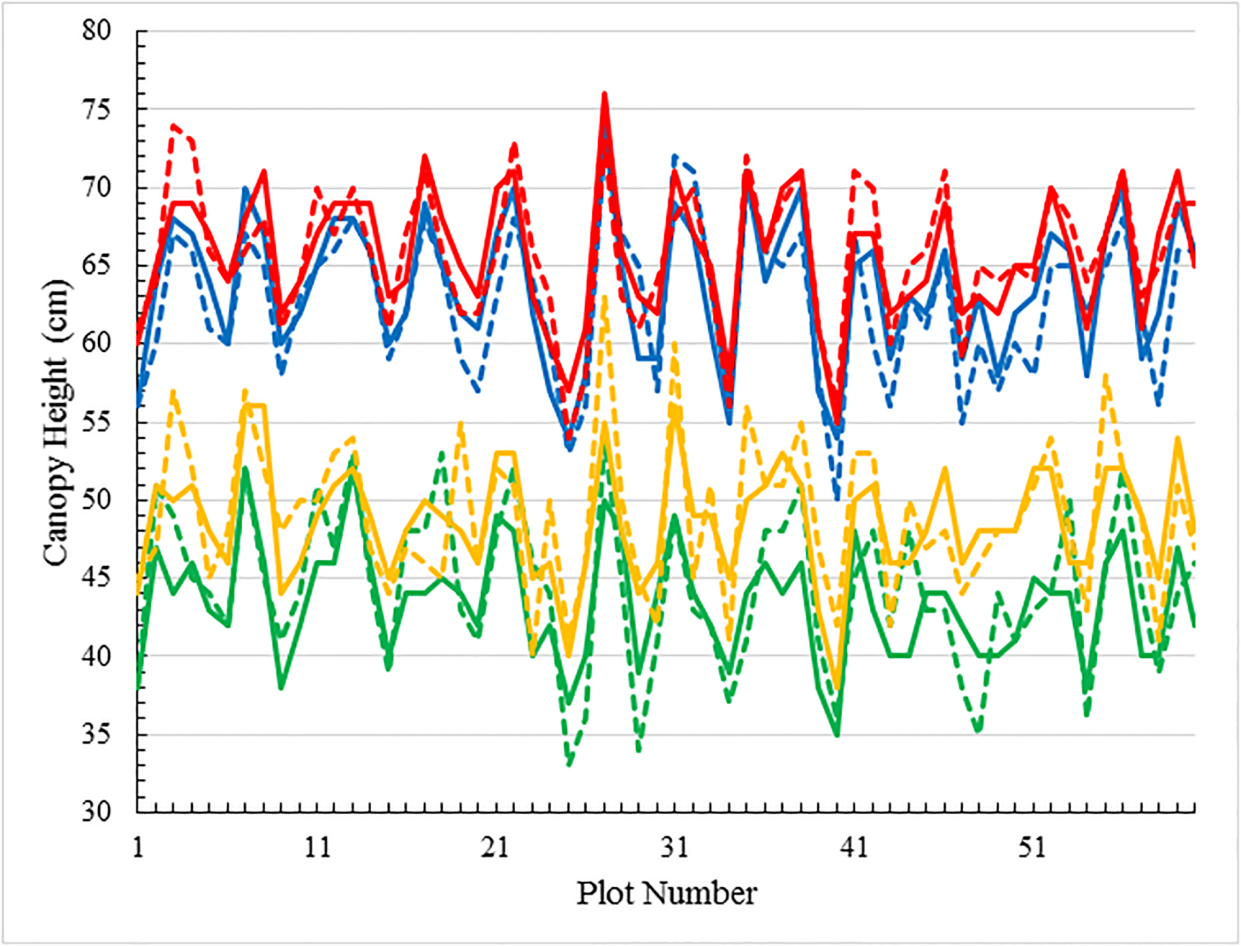
Graphical comparison of canopy heights for different days. Graphs of manual measurements (dashed lines) and the proposed depth map estimations (solid lines) of canopy height for the complete data set comprising 60 plots and 4 days of results. The four colors represent the data for the four different days: 23/09/2016 (green), 27/09/2016 (yellow), 11/10/2016 (blue) and 14/10/2016 (red).

Fig 3 summarizes the strong correlation present between the theoretical height estimations and the manual measurements on different days (pre-spike appearance). A somewhat more detailed comparison as a function of individual plots is shown in Fig 4. Generally, heights estimated by stereo matching are close to those obtained manually. Using these manual measures as benchmark, the standard variance of the automated height estimation from ground truth is found to be 2.9*cm*. Having said this, the reference to the manual measurements as a “benchmark” should be considered with a degree of some scepticism. It was explained earlier that the recorded manual measurements were the results of averaging of a number of height samplings. This protocol presumes that the ground is even (which is unlikely since the planting process creates furrows resulting in an uneven, indeed undulating, ground base) and that the canopy is uniform (which is unlikely due to edge effects and possibly also to soil variability across the plot). Consequently, it should be appreciated that some degree of error or variability is inherent in the benchmark itself.

**Fig 5:**
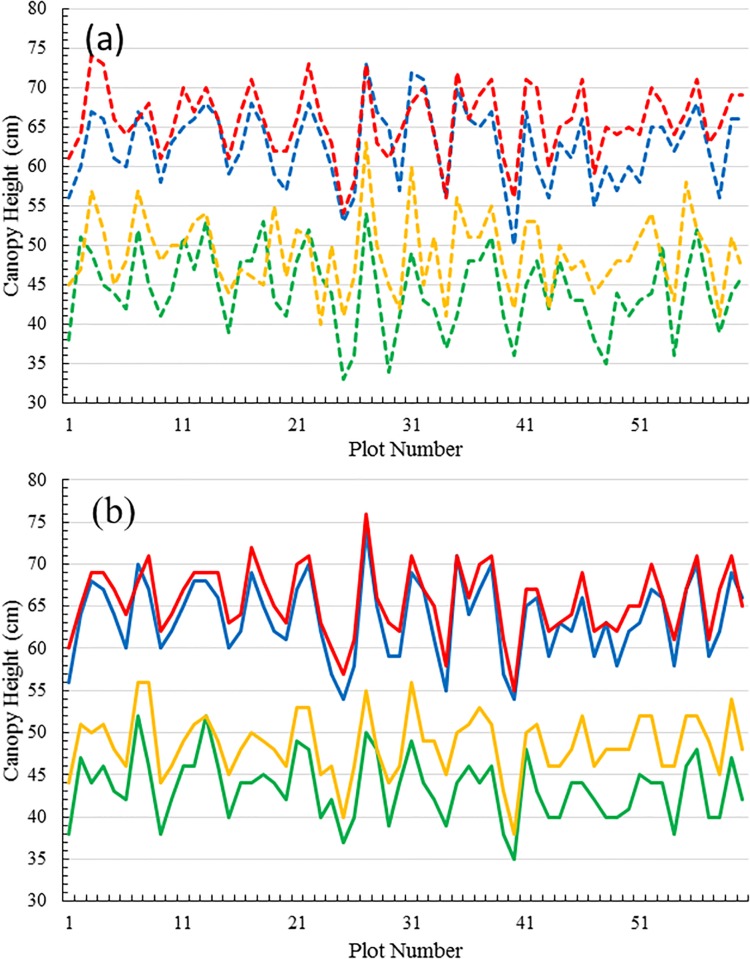
Graphical comparison of canopy heights for a self-consistency study. Graphs of canopy height for the complete data set as per Fig 4, except that manual measurements and theoretical estimations are separated for clarity of discussion. (a) Plant heights as obtained by manual measurement; (b) plant heights obtained by stereo matching. As above, the four colors represent data from the four imaging days: 23/09/2016 (green), 27/09/2016 (yellow), 11/10/2016 (blue) and 14/10/2016 (red).

To highlight this variability we consider in Fig 5 the same data as in Fig 3 but separated for clarity into manual measurements (Fig 5(a)) and theoretical estimations (Fig 5(b)). The curves again represent the state of the plots on the four imaging days: 23/09/2016, 27/09/2016, 11/10/2016 and 14/10/2016. Given the time differences, one would expect there to be a significant difference in height between the data for the first pair of days and the data for the second pair of days, which is indeed realized in both figures. However, although less difference would be expected within each pair, which differ in time by only four and three days, respectively, it is not always realized that plant heights on the second day of each pair are higher than on the first day. Fig 5(a) indicates 12 exceptions, while Fig 5(b) indicates only two exceptions. In the former case, the exceptions likely originate in either subjective errors made in measurements of canopy height or in the variation of ground height where the ruler was positioned. As for the automated height estimation by stereo matching, the method has two major advantages over manual measurements. Firstly, the estimation is always objective, and secondly the ground level reference is a constant. These facts contribute to the improvement over manual determination. All the same, automated height estimation is not a perfect process and relies on accurate stereo matching to produce accurate depth maps. The two exceptions shown in Fig 5(b) are likely due to disparities in the stereo matching process. In relative terms, however, the favourable comparison of self-consistency of Fig 5(b) over Fig 5(a), advocates for the more objective approach to height determination as provided by our automated method.

### Canopy height distributions as phenotypic traits

Plant height is without doubt one of the more important traits used for plant phenotyping purposes. It has been used for crop lodging detection [2] and has been included as a component in biomass estimation [3, 17]. In this section we demonstrate that far greater understanding of canopy development is possible from the information contained in depth maps rather than just the canopy height.

**Fig 6:**
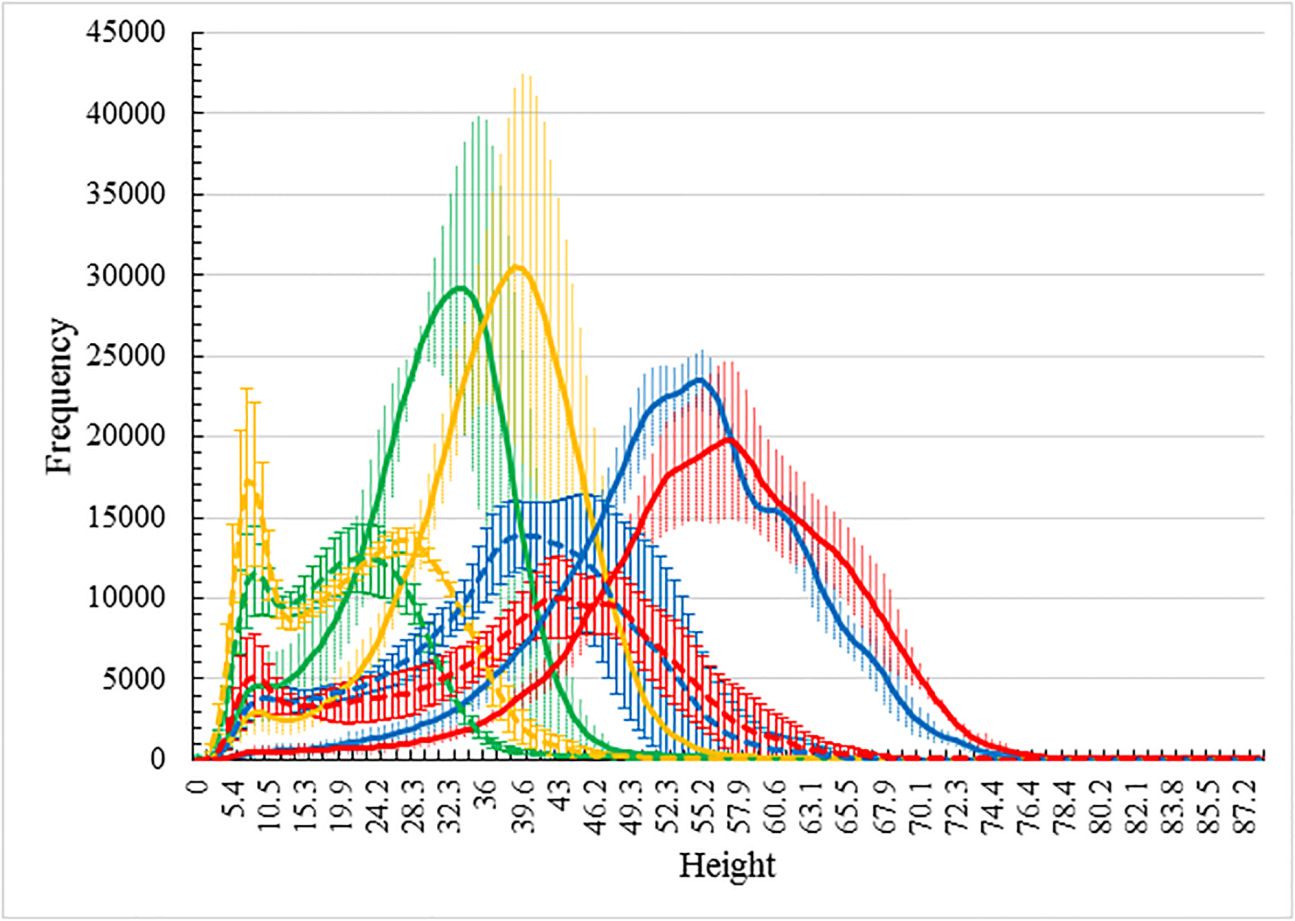
The effect of treatment on height distribution. Demonstration of the effect of treatment on canopy height distribution as registered by differences in frequency histograms of leaf pixel height for the four days of imaging: 23/09/2016 (green), 27/09/2016 (yellow), 11/10/2016 (blue) and 14/10/2016 (red). The Australian wheat cultivar Drysdale was chosen for study due to its pronounced dependence on canopy fertilization. The solid lines for each respective day refer to averages of the three replicate fertilized Drysdale plots (error bars show variation over the three repeats). The dashed lines for each respective day refer to averages of the three replicate *un*-fertilized Drysdale plots (error bars show variation over the corresponding three repeats).

One particular value that depth maps add to our understanding of coverage and leaf distribution at depth becomes highlighted when depth maps for canopies of different varieties or different treatments are compared. For example, Fig 6 compares depth histograms of the Australian wheat variety Drysdale under two treatments, with (solid lines) and without (dashed lines) fertilizer, for the four imaging days during the season. Each line represents the mean of the three corresponding replicates. The variations across those replicates are shown as error bars (color coded for the respective days, and dotted and solid error bars, respectively, for treated and untreated plots). The variety Drysdale was singled out for illustration as it appeared to be the variety out of the ten studied that was most responsive to nitrogen treatment. From this figure, a number of features are apparent.

Firstly, from 23/09/2016 to 14/10/2016, the plants in all plots grew, as measured by height and canopy coverage. Secondly, plants in the fertilized plots grew significantly taller with thicker canopies than plants in the unfertilized plots. Thirdly, comparing the average histograms for fertilized plots for day 23/09/2016 with those for day 27/09/2016 one can see that the whole histogram average has shifted which can be explained by stem elongation, which resulted in the leaf canopy being raised further above the ground, without an accompanying change in the shape of the distribution. This is demonstrably less so, but still arguably the case, for the unfertilized plots. Similar statements describing the change in the histograms from day 11/10/2016 to 14/10/2016 cannot be made as internal adjustments in the distribution occur alongside general height extensions. From these histograms, we also find that canopy coverages (the areas under histogram curves) increase significantly during the 4 day period from 23/09/2016 to 27/09/2016.

A canopy distribution also allows for a refined understanding of other canopy changes: the canopy thicknesses on 14/10/2016 were lower than those on 11/10/2016 even though plant heights increased slightly. This we understand to be the result of some plant senescence. That is, we find that the upper portions of histograms are very similar, while the lower portions are quite different: frequencies of the lower parts decreased significantly. This attributed to the wilting and volume loss in the lower plant shoots following senescence which commonly starts in the lower parts of plants. The canopy height distribution is thus useful in not only providing information about growth overall but also the existence of some canopy senescence.

**Fig 7:**
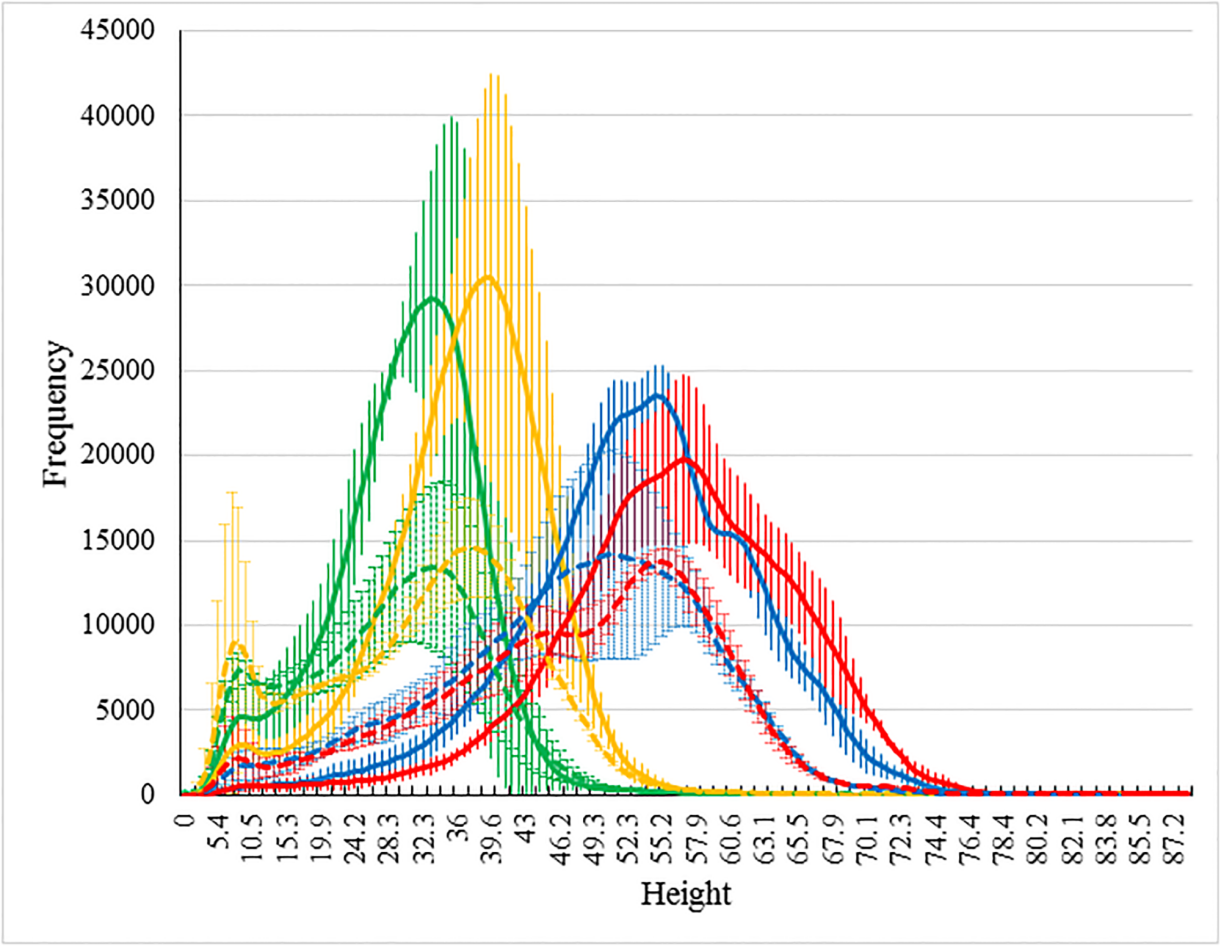
Comparison of canopy development for two varieties: Drysdale and Mace. Demonstration of the different rates and extents of development of canopy for two Australian cultivars as captured by their leaf pixel height frequency histograms, as a function of time. The colour coding again refer to the four days of imaging: 23/09/2016 (green), 27/09/2016 (yellow), 11/10/2016 (blue) and 14/10/2016 (red). The Australian wheat cultivars Drysdale and Mace were chosen as these exhibited considerable differences in mean canopy height. Only data from fertilized plots is included in the analysis. The solid lines for each respective day depict averages of the three replicate fertilized Drysdale plots (error bars show variation over the three repeats). The dashed lines for each respective day depict averages of the three replicate fertilized Mace plots (error bars show variation over the corresponding three repeats). Note that in this study in contrast to Drysdale (Fig 6), Mace did not show any significant variation with fertilizer.

On the matter of varietal differences, Fig 7 shows depth map histograms for the varieties Drysdale and Mace. The results are for fertilizer-treated plots. As in Fig 6 the mean values are shown as continuous lines with error bars indicative of the variation across the respective three replicates; color codes again reflect the four different days of imaging. The varieties have been selected for illustration for their large relative difference in growth and development patterns. The Drysdale results are identical to those shown in Fig 6. We point out that the canopy height distributions for fertilized and unfertilized plots of Mace, in contrast to Drysdale, did not show any major structural differences.

In summary, Fig 7 shows that Drysdale (solid lines) performed better in terms of both canopy coverage and height on all four occasions, although the height difference between Drysdale and Mace is less obvious. The is probably the result of Drysdale’s breeder selection as a water efficient variety, which is an important characteristic for wheat plants growing in non-irrigated fields. In contrast, the rationale behind Mace’s selection was its ability to perform well without fertilizer. If we consider the results in finer detail, we see that Drysdale performed better in terms of canopy coverage but with similar heights on 23/09/2016 and 27/09/2016, whereas on 11/10/2016 and 14/10/2016 Drysdale performed better on both counts. The situation for the first two days of imaging is somewhat atypical in that plant height is usually closely correlated with plant canopy coverage. This may have implications for predictive applications. For example, Bendig et al. [17] proposed a method to estimate plant biomass based on canopy height and vegetation indices. Their biomass model would give consistent results in cases where plant height was closely correlated with canopy coverage since vegetation indices have been widely used in studies of senescence. However, the atypical behavior indicated by the height distributions in Fig 7 clearly indicate that canopy height in itself is not always a reliable measure for biomass estimation.

From both Figs 6 and 7, one can conclude overall that canopy height distributions can reveal both subtle as well as not so subtle differences in growth trends for different wheat varieties and for different treatments.

**Fig 8:**
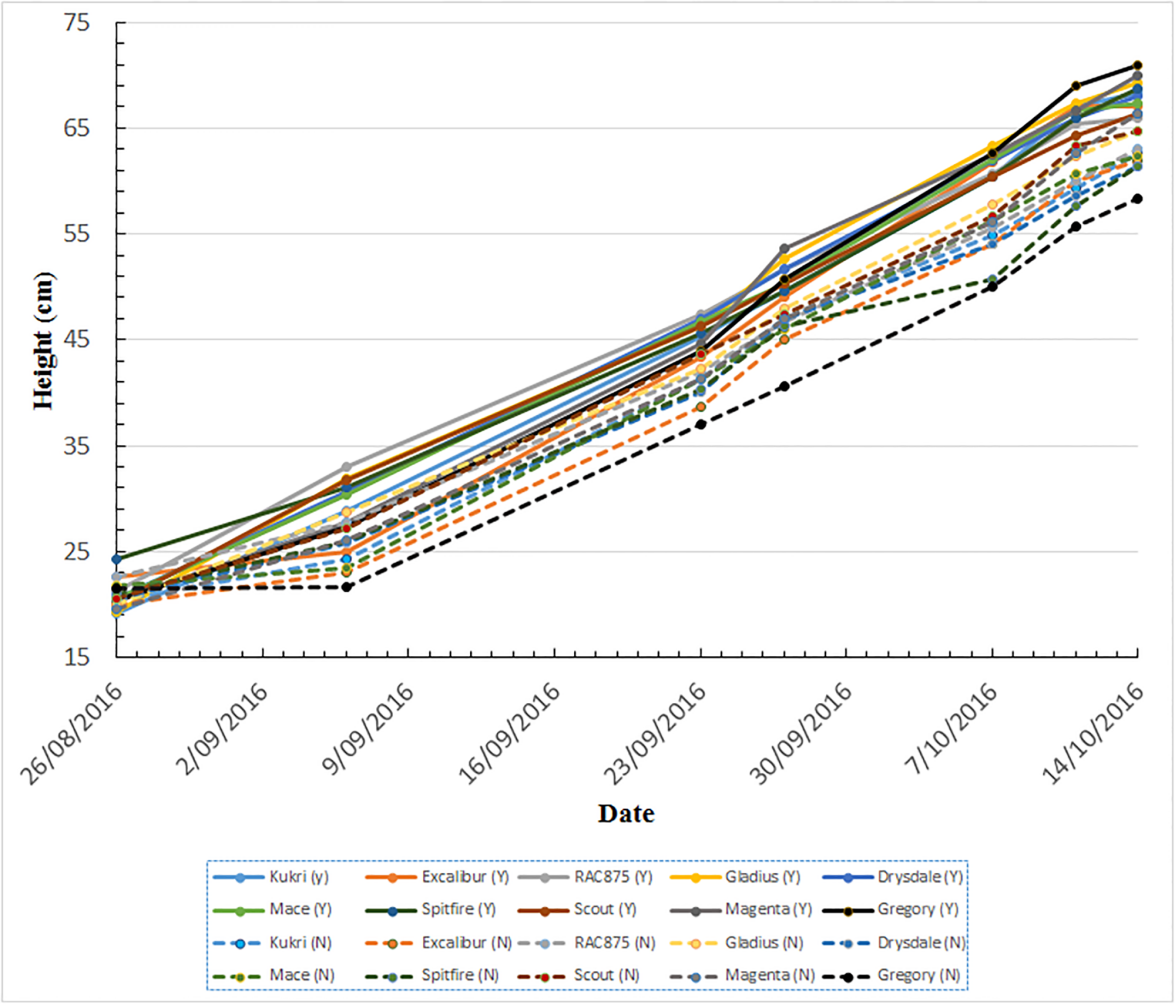
The overall growth behaviour of all 10 wheat varieties under two different fertilizer treatments. Y: fertilized (solid lines) and N: without fertilizer (dashed lines).

With regard to the growth behaviour as a function of time, Fig 8 depicts the growth in term of height of all 10 wheat varieties under the two different fertilizer treatments, from 26/10/2016 to 14/10/2016. The effects of fertilizer treatment on all 10 wheat varieties, in terms of plant canopy height, are obvious from 06/09/2016. All varieties under fertilized treatment are clearly taller than these without fertilizer treatment. It is also clear that the plant canopies exhibited almost linear growth with time, for both fertilizer treatments from 06/09/2016 to 11/10/2016. It is also interesting to note that growth slowed down just before the heading growth stage as evidenced by the canopy heights from 11/10/2016 to 14/10/2016.

**Fig 9:**
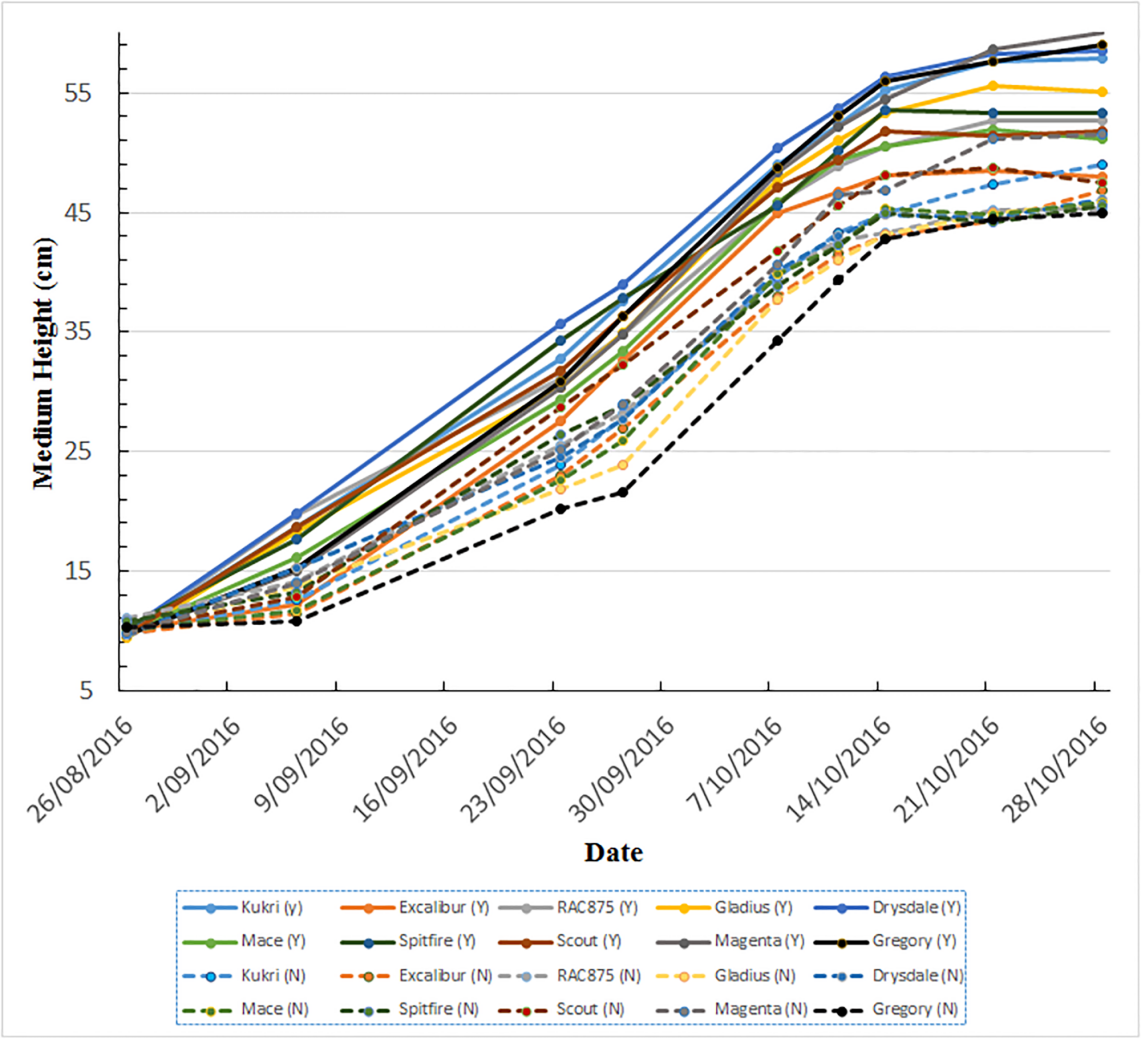
Comparison of plant development as a function of time in terms of medium canopy height under the two fertilizer treatments for all varieties. Y: fertilized (solid lines) and N: without fertilizer (dashed lines).

In the approach proposed here, the overall canopy height is estimated by thresholding the top 2% of the depth map histograms. The advantage of this approach is that canopy height estimations are consistent with manual measurements. However, the accuracy of the canopy height estimation is sensitive to spike appearance as illustrated in Fig 2. As an alternative, it is reasonable to use the medium canopy height, which can be estimated by thresholding the top 50% of the depth map histograms, as one of phenotypic traits. The major advantage of the medium canopy height is its robustness to small variations in depth map histograms caused by spikes. Fig 9 shows the medium canopy heights of plants under two different fertilizer treatments from 26/08/2016 to 28/10/2016. By comparing the Figs 8 and 9, one can see that both canopy height measures exhibit similar growth behaviours from 06/09/2016 to 14/10/2016. Extending the observations by another two weeks in which time spikes appear we find that the medium canopy heights are maintained at relatively the same levels, despite a height increase of plants due to the presence of spikes.

**Fig 10:**
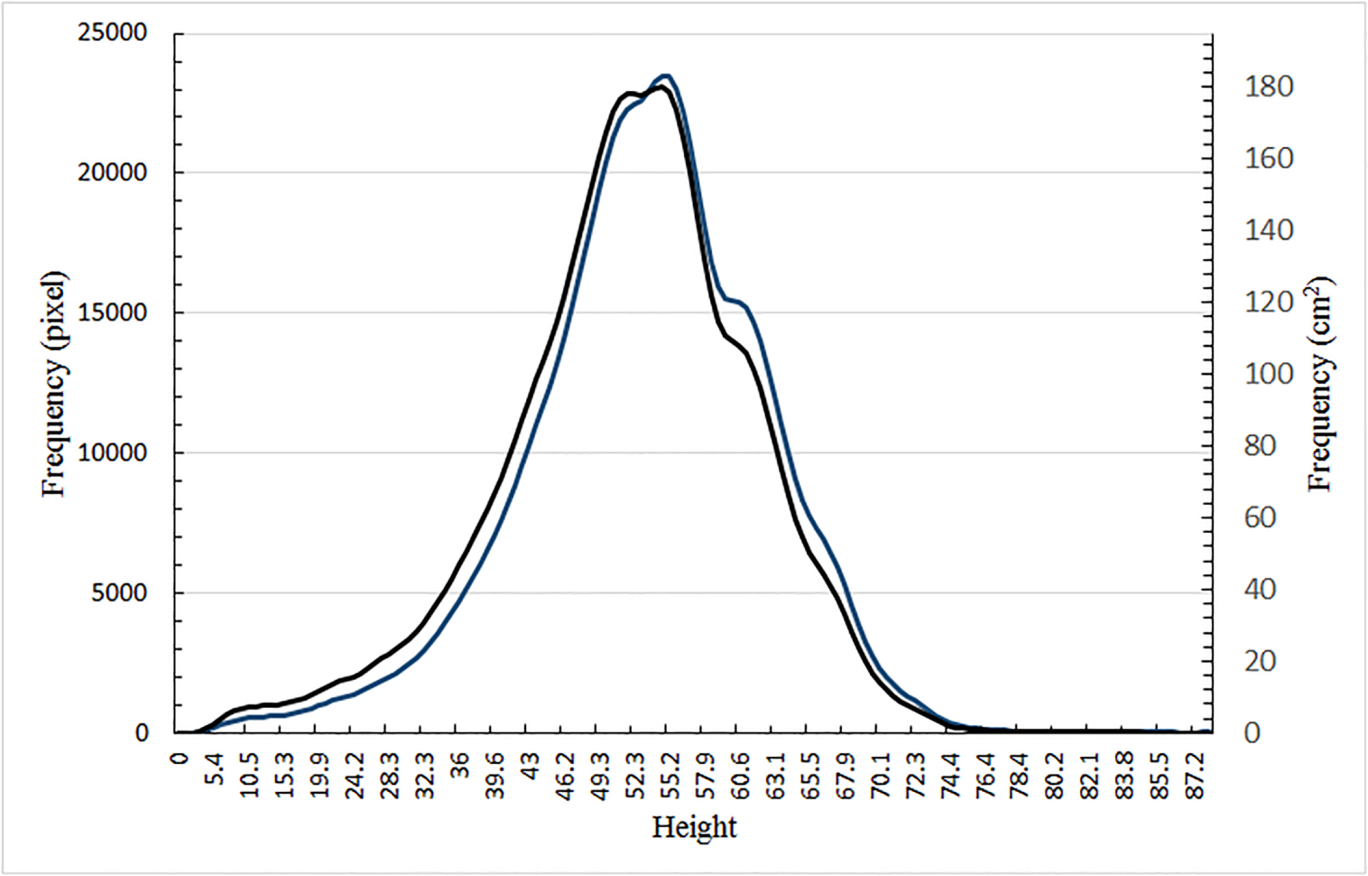
Normalization of leaf height frequency histograms. Comparison of frequency histograms between un-normalized and normalized canopy height distributions. Normalization, allowing comparisons with different hardware configurations, involves a conversion to leaf pixel distribution (un-normalized, blue line and left vertical axis) to leaf area distribution (normalized, black line and right vertical axis).

Finally, we make the observation that since camera settings may differ for different imaging platforms, it is important to provide an absolute basis for a quantitative comparison. For example, a bigger image of a plant leaf will result for a camera that is closer to the leaf than for a camera that is further away. A difference in distance will mean that the resolution of the ground will also be different from that of the plant leaves. Similarly, the resolution of plant leaves at different heights will also differ. Consequently, an analysis can either over-estimate or under-estimate the actual plant canopy coverage, depending on camera position. To overcome this complication one can generate normalized depth map histograms in which the frequency unit refers to (dimensional) leaf area as opposed to image pixel frequency. A comparison between normalized and un-normalized histograms is shown in Fig 10.

It is worthwhile reflecting at this point how such normalized canopy height distributions may feature in the definition of plant biomass. It is known that the different above-ground tissues of a given plant have different mass densities due to their different cellular compositions—in wheat we restrict attention to the major differences between the leaf, the stem and the spike tissues. Assuming no dependence on plant position, we can denote their wet-weight densities *ρ*_*lf*_, *ρ*_*st*_ and *ρ*_*sp*_, respectively. Suppose now that the normalized leaf, stem and spike area distributions, as a function of height, are *a*_*lf*_(*h*), *a*_*st*_(*h*) and *a*_*sp*_(*h*), respectively. The plant biomass of a given plot, *M*_*bio*_, can then be defined as

$$\begin{array}{c} M_{bio}=\int_{0}^{T}\rho_{lf}a_{lf}\left(h\right)dh+\int_{0}^{T}\rho_{st}a_{st}\left(h\right)dh+\int_{0}^{T}\rho_{sp}a_{sp}\left(h\right)dh. \end{array}$$ (1)

where all three contributions are expressed in the form of integrals over height from the ground level (*h* = 0) to the absolute top of the canopy (*h* = *T*). Thus, apart from the wet-weight densities, we see that for a true estimate of (wet) biomass one needs to know the respective tissue areas as a function of height. Taking the rather debatable approximation of constant and equal tissue densities, *ρ*, the above expression simplifies to

$$\begin{array}{c} M_{bio}=\rho \int_{0}^{T}(a_{lf}\left(h\right)+a_{st}\left(h\right)+a_{sp}\left(h\right))dh. \end{array}$$ (2)

Our normalized canopy height distribution captures the sum total argument of the integrand. The integral itself is the area under the normalized distribution shown in Fig 10. Consequently, a proper estimation of biomass requires just the normalized distribution that we have determined using stereo images. In contrast, the typical approach involves the top view of canopy coverage, which we denote as, *A*, and canopy height, *T* giving the product *ρAT*, which is a significant overestimation of the actual canopy biomass (it conveys the idea of a uniform rectangular block of plant material). If possible this simple product is to be discouraged in preference to Eq (2).

## Conclusions

On the practical side, we have demonstrated that our effective and novel camera self-calibration approach can facilitate the extraction of quality depth maps from stereo images. Moreover, we have shown that from these depth maps one can accurately extract canopy heights and canopy height distributions as well as other phenotypic information. The main advantage of the self-calibration method is that it does not require any image processing knowledge nor any additional calibration steps. The user is only required to obtain a stereo image of a relatively flat area of ground at the time of imaging. The method is particularly suitable for field applications.

The results of our study, based on a sequence of images taken of 60 plots in a field experiment involving 10 wheat varieties subjected to two different fertilizer treatments, have compared favorably with manual measurements of canopy heights, indeed to a high degree of high accuracy (an R-squared value of 0.92). In fact, the level of consistency based on our automated approach is far superior to the level of consistency possible by manual means. The few deviations from consistency were attributed to minor discrepancies in the stereo matching step. In contrast, manual measurements were found to exhibit a greater number of inconsistencies, which we attributed to the subjective nature of the method used. An important conclusion to draw from the comparison is the potential for a more robust and more objective means of quantifying this important phenotypic trait.

A significant outcome of our comparisons of canopy height distributions (for different varieties subjected to the same treatment as well as for the same variety subjected to different treatments) is the potential with this quantity to now reveal more detail about the different extents to which plants respond to growth conditions. From our analysis it may no longer be clear that a simple overarching trait such as canopy height is sufficient to describe all aspects of growth behavior.

Canopy height and even a canopy height distribution are not the sole nor sufficient means of characterizing crop growth and health. Other traits are needed. However, to determine these in a field setting is challenging for a variety of reasons. The most obvious factors are variable weather and lighting conditions, the considerable spatial extents to be covered, but also the fact that plants in fields are grown in close proximity resulting in considerable occlusion thus preventing direct observation. In this paper we have focused attention on a better estimation of one trait. However, it is possible to take further advantage of the 2D and 3D information obtained from RGB images to derive other quantitative phenotypic information. In separate studies, for example, we address the variable lighting issue, biomass estimation and wheat spike identification. Many of these features can be obtained from 3D information which we have shown is possible to obtain using high-resolution images from a land-based imaging platform. LiDAR can also provide 3D canopy information [12]. However, it is difficult to integrate 2D images with 3D information from LiDAR sensors in the field due to the constant and irregular movement of plant leaves during the LiDAR scanning process by the action of wind. Simple synchronized RGB stereo imaging methods such as the one employed here, on the other hand, can avoid such problems. To further exploit the merits of a land-based imaging system, we are developing a novel algorithm for plant segmentation and plant senescence analysis that does not rely on complicated numerical methods such as machine learning, which usually requires tedious manual labelling during a training process [23].

## Methods

### Stereo matching

**Fig 11:**
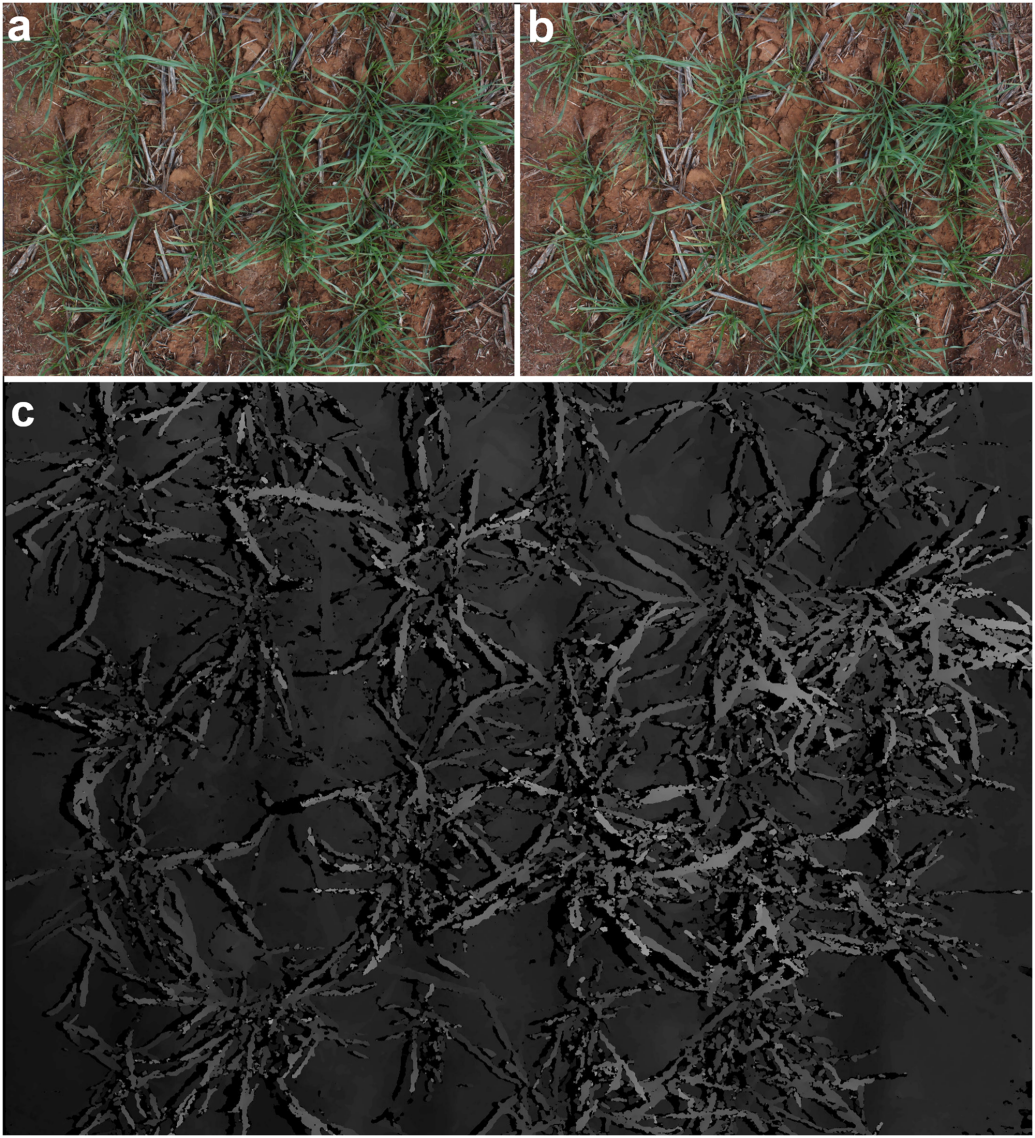
Image rectification and depth map. Top: A stereo image pair taken of plot 57 on day 26/08/2016. The rectified image from the left camera is shown in (a) and the rectified image from the right camera is shown in (b). Bottom (Fig (c)): The resulting depth map.

After self calibration and lens distortion correction as described in the supporting document, we can rectify the images to create stereo image pairs and this process is called image rectification. In this study, Hartley’s algorithm [26] is implemented in our procedure for image rectification due to its robustness. With stereo image pairs, we can estimate the disparity between pairs of stereo images and create depth maps. Semi-global matching [27] is an efficient strategy for approximately minimizing a global energy that comprises a pixel-wise matching cost and pair-wise smoothness terms. Due to its performance in terms of speed and accuracy, we apply this algorithm to estimate disparity from stereo image pairs and then generate depth maps. Some examples of rectified images and their corresponding estimated depth map are shown in Fig 11.

Once the depth maps of field plots have been generated, they are converted to height distributions. The first step of the plot height estimation procedure is to estimate the heights of individual pixels in a depth map. These are then used to generate a height histogram, which is then used to generate the canopy height distribution of plants and estimate the representative plot height. The disparity *d*_*xy*_ of a point P at (*x*, *y*) in a depth map can be converted to a depth *P*_*xy*_ in a conventional computer vision sense [28]. The depth *P*_*xy*_ can be calculated using

$$\begin{array}{c} P_{xy}=\frac{fG}{d_{xy}S_{c}}, \end{array}$$ (3)

where *P*_*xy*_ is the depth between *P* in real world and camera apertures, *f* is the effective focal length, G is the distance between camera apertures illustrated in Fig 1 and *S*_*c*_ is the pixel size of the camera sensor. This assumes that camera apertures are at zero depth. The height of the point above the ground level can be obtained by

$$\begin{array}{c} h_{xy}=H-P_{xy}, \end{array}$$ (4)

where *H* is the camera height above ground level.

In our approach we propose to treat the ground level as zero height. The plant height at (*x*, *y*) is then

$$\begin{array}{cc} h_{xy}= & \frac{H^{2}\eta_{xy}S_{c}}{fG+(\eta_{xy}S_{c}H)}, \end{array}$$ (5)

$$\begin{array}{cc} \eta_{xy}= & d_{xy}-d_{g}, \end{array}$$ (6)

where *η*_*xy*_ is the normalized disparity and *d*_*g*_ is the average disparity of the ground level. The use of Eq 6 has one major advantage over the use of Eq 4, that is the robustness to the vibration of the phneotyping platform. Unlike a laboratory environment, it is inevitable that there is vibration when the platform is operated in filed environment. Usually the vibration can cause small errors in disparity estimation and it transmits to the depth estimation errors if Eq 4 is used. However, these errors will cancel out in Eq 6 as vibrations cause the same error in *d*_*xy*_ and *d*_*g*_ in the same depth map. Therefore, our approach has a practical advantage in field conditions over the approach by Szeliski [28], which is widely used in the computer vision community and is designed for laboratory conditions.

**Fig 12:**
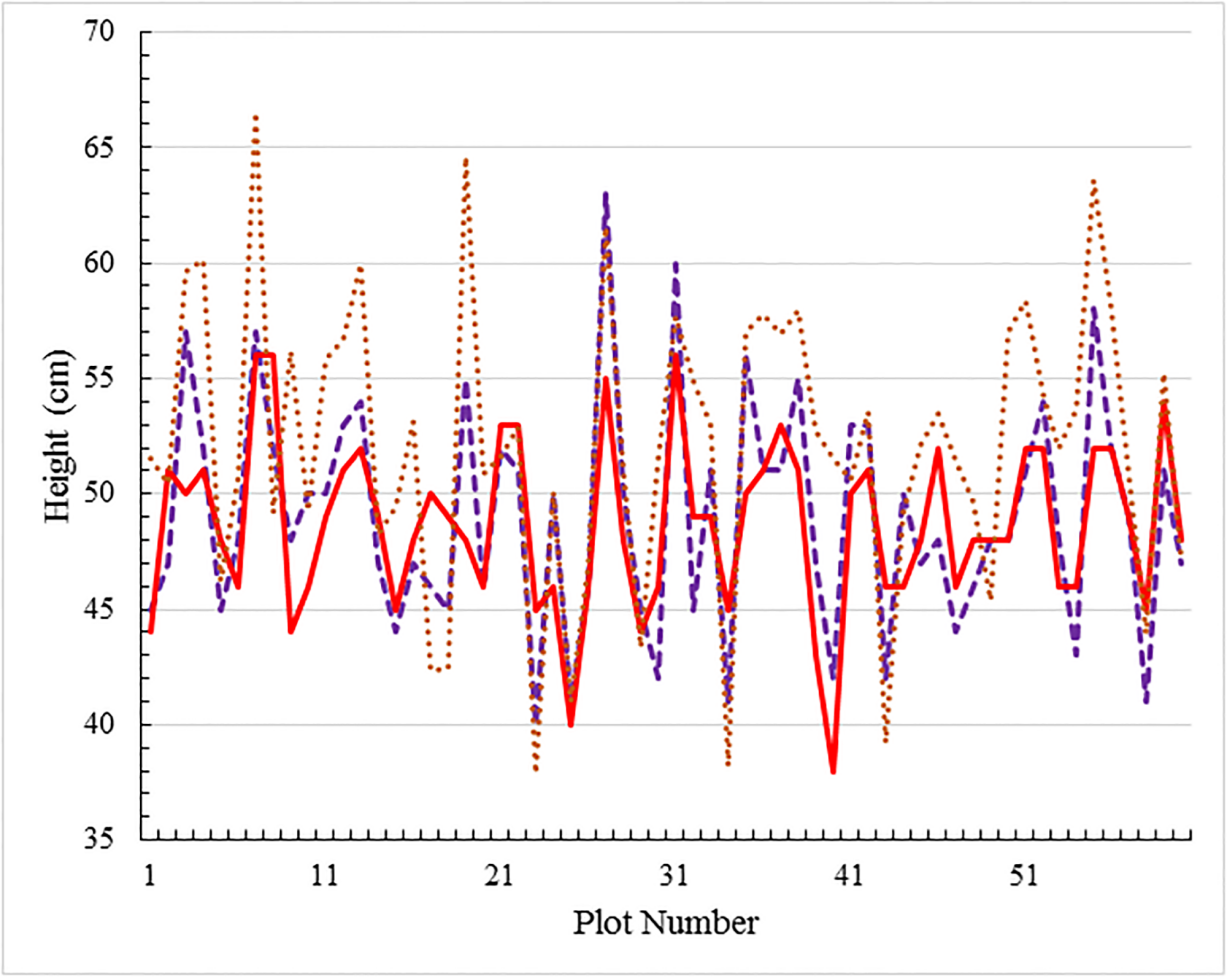
Comparison of height estimation techniques. A single time stamp (27/09/2016) comparison of different methods of canopy height estimation for the 60 plot set of ten wheat varieties, two treatments and three replicates. The solid red line represents the heights estimated by the proposed automated method based on stereo images and derived depth maps. The dashed line depicts heights obtained by manual measurement, while the dotted line represents canopy heights estimated from images that have been calibrated using a more conventional approach that relies on additional reference data.

In Fig 12 we compare the height estimation results based on the method of calibrating camera images using the stereo pair method proposed here, with results based on a conventional camera image calibration method [29, 30], which uses speeded up robust features (SURF) [31]. for pattern recognition and requires multiple images of specifically designed patterns for a reference. Also included are the manual measurements taken as described in the paper. Given that the difference between the two theoretical sets of results lies in the method applied to calibrate camera images, the agreement between all three approaches is good. This provides further support for the approach proposed in this paper as an effective method of accurate height estimation.

## Supporting information

S1 File Camera self-calibration is achieved by three steps: Lens distortion modelling, the process of self-correction and the optimization. (PDF)
