# Supplementary material for: Land-based crop phenotyping by image analysis: Accurate estimation of canopy height distributions using stereo images
Source: PLoS One. 2018 May 24;13(5):e0196671. doi: 10.1371/journal.pone.0196671 (PMC5967702; doi:10.1371/journal.pone.0196671)
Supplement: S1 File — (PDF) [file pone.0196671.s001.pdf]

# Land-based crop phenotyping by image analysis: accurate estimation of canopy height distributions using stereo images

Jinhai Cai, Pankaj Kumar, Joshua Chopin, Stanley J. Miklavcic

## Camera self-calibration

### Lens distortion

Camera calibration has been a very important issue to deal with in many applications [1], particularly in machine vision where cameras, such as Canon 60D and 70D, with zoom lenses are used. A zoom lens comprises an assembly of various lens elements to allow for a range of focal lengths, it is inevitable that some distortion is introduced in images. With the improvement of lens manufacturing techniques, the problem of image distortion caused by zoom lenses has been significantly alleviated but not solved completely. Usually, lens distortion can be classified as radial distortion. Lens distortion can not only reduce the visual quality of images but also have a significant impact on the accuracy of 3D reconstruction of objects [1]. In order to reduce the impact of radial distortion in computer vision applications various mathematical models of image distortion and methods have been proposed. Pioneer work in image distortion modelling and camera calibration has been described by Brown in [2], in which a real camera is represented as an ideal (*i.e.*, pinhole) camera with lens distortion. Since then Brown's distortion model has been widely adopted in the

computer vision community. The equations intrinsic to Brown's model are:

$$\begin{aligned}\hat{x} &= x + (x - x_c)(K_1 r^2 + K_2 r^4 + ...) + \\ & [P_1(r^2 + 2(x - x_c)^2) + 2P_2(x - x_c)(y - y_c)] \\ & (1 + P_3 r^2 + ...) \qquad \text{(S1)} \\ \hat{y} &= y + (y - y_c)(K_1 r^2 + K_2 r^4 + ...) + \\ & [P_2(r^2 + 2(y - y_c)^2) + 2P_1(x - x_c)(y - y_c)] \\ & (1 + P_3 r^2 + ...) \qquad \text{(S2)}\end{aligned}$$

where $(\hat{x}, \hat{y})$ is a point in an undistorted image, $(x, y)$ is a point in a distorted image, $(x_c, y_c)$ is the distortion centre (*i.e.*, the principal point), $K_n$ is the $n$th radial distortion coefficient, $P_n$ is the $n$th tangential distortion coefficient, and $r = \sqrt{(x - x_c)^2 + (y - y_c)^2}$. Usually, both $K_n$ and $P_n$ are called distortion parameters.

Rahman and Krouglicof [3] investigated the robustness and accuracy of Brown's model for a wide range of lens distortions. They found that, for most camera except web camera, a lower order lens distortion model produces satisfactory accuracy at considerable computational saving [3]. In practice, we can achieve satisfactory performance for radial distortion correction using a Brown's model with just first or second order radial distortion coefficients.

There have been many algorithms developed for the estimation of model parameters. In one class of methods [2, 3], the relationship between 3-D reference coordinates and 2-D image coordinates is required. The estimation of the camera model parameters is then achieved numerically according to the set of the 3-D to 2-D correspondences. The major advantage of this approach is that extrinsic and intrinsic camera parameters and distortion parameters can be estimated simultaneously. However, obtaining accurate coordinates of 3-D scene points is sometimes demanding [4]. Usually, a calibration object of known structure is used for extracting accurate coordinates of 3-D scene points. A second class of methods [5] exploit the regular patterns of planar objects such as a chessboard for camera calibration. Zhang [5] used multiple views of planar grids taken from unknown viewpoints to estimate lens distortion parameters as well as camera parameters. In a third class of methods, no calibration object is required. Some

methods [4, 6] estimate lens distortion parameters from individual images by line rectification. Alvarez and Sendra [6] proposed an algebraic approach for the estimation of lens distortion parameters by line rectification, in which lines are predetermined by manual selection. Ahmed and Farag [4] proposed to use a robust approach based on the least-median-of-squares (LMedS) for the estimation of lens distortion parameters (one radial distortion coefficient and one tangential distortion coefficient). In theory, the LMedS method can handle up to 50% outliers in the input data. Therefore, their method [4] can obtain reasonable estimates if most extracted curves are straight lines in the undistorted images.

Unfortunately, in many applications, such as the field imaging situation currently under discussion, there are no or too few straight lines that can be captured in images. Therefore, the methods described above cannot be employed for plant crop phenotyping. Farid and Popescu [7] developed a method based on spectral information to estimate the lens distortion model parameters. However, the accuracy with which this was obtained was compromised because only substantial lens distortion can be detected in spectral information. In this paper, we take full advantage of the use of stereo cameras to develop a robust self-calibration algorithm that does not rely on peripheral hardware such as a calibration chart.

## The process of self-correction for lens distortion

For a given stereo pair of cameras, the cameras are positioned with optical axes aligned in one plane. In this application to field phenotyping we exploit the fact that crops are planted on locally flat fields to achieve self-calibration. If the lenses of stereo cameras are undistorted and the plane of the camera sensors is parallel to the ground plane, the distance between any two points on the ground (plane) in the right image will be the same as the distance between the two points detected in the left image taken by the stereo camera pair. Let $P_1$ and $P_2$ be two arbitrary points in the ground plane. We then have that

$$(u_{I_1} - u_{I_2})^2 + (v_{I_1} - v_{I_2})^2 = (u_{J_1} - u_{J_2})^2 + (v_{J_1} - v_{J_2})^2, \tag{S3}$$

where $(u_{I_1}, v_{I_1})$ and $(u_{I_2}, v_{I_2})$ are the coordinates of $P_1$ and $P_2$, respectively, in image $I$; and $(u_{J_1}, v_{J_1})$ and $(u_{J_2}, v_{J_2})$ are the coordinates of $P_1$ and $P_2$, respectively, in image $J$.

It is well known that zoom cameras deviate from the ideal pinhole model. Fortunately, in most cases, the first-order Brown's model can adequately correct for this distortion caused by the camera lenses. In such a case, the first-order Brown's model gives

$$u - x_0 = (\hat{x} - x_0)(1 + k * r^2), \tag{S4}$$

$$v - y_0 = (\hat{y} - y_0)(1 + k * r^2), \tag{S5}$$

where $(\hat{x}, \hat{y})$ and $(u, v)$ are the coordinates of a point in the original image and in the distortion corrected image, respectively. The distortion centre is denoted $(x_0, y_0)$, $r^2 = (\hat{x} - x_0)^2 + (\hat{y} - y_0)^2$, and $k$ is the first-order distortion parameter. For convenience, let $x$ denote $\hat{x} - x_0$ and $y$ denote $\hat{y} - y_0$. By applying (S4) and (S5) to (S3), we have

$$\begin{aligned} &(x_{I_1} - x_{I_2})^2 + (y_{I_1} - y_{I_2})^2 + 2k(x_{I_1} - x_{I_2})(x_{I_1} r_{I_1}^2 - x_{I_2} r_{I_2}^2) \\ &+2k(y_{I_1} - y_{I_2})(y_{I_1} r_{I_1}^2 - y_{I_2} r_{I_2}^2) \\ &+k^2[(x_{I_1} r_{I_1}^2 - x_{I_2} r_{I_2}^2)^2 + (y_{I_1} r_{I_1}^2 - y_{I_2} r_{I_2}^2)^2] \\ &= (x_{J_1} - x_{J_2})^2 + (y_{J_1} - y_{J_2})^2 + 2k(x_{J_1} - x_{J_2})(x_{J_1} r_{J_1}^2 - x_{J_2} r_{J_2}^2) \\ &+2k(y_{J_1} - y_{J_2})(y_{J_1} r_{J_1}^2 - y_{J_2} r_{J_2}^2) \\ &+k^2[(x_{J_1} r_{J_1}^2 - x_{J_2} r_{J_2}^2)^2 + (y_{J_1} r_{J_1}^2 - y_{J_2} r_{J_2}^2)^2]. \end{aligned} \tag{S6}$$

We rewrite this equation as

$$Ak^2 + Bk + C = 0, \tag{S7}$$

with

$$\begin{aligned} A &= [(x_{I_1} r_{I_1}^2 - x_{I_2} r_{I_2}^2)^2 + (y_{I_1} r_{I_1}^2 - y_{I_2} r_{I_2}^2)^2] - [(x_{J_1} r_{J_1}^2 - x_{J_2} r_{J_2}^2)^2 + (y_{J_1} r_{J_1}^2 - y_{J_2} r_{J_2}^2)^2], \\ B &= 2(x_{I_1} - x_{I_2})(x_{I_1} r_{I_1}^2 - x_{I_2} r_{I_2}^2) + 2(y_{I_1} - y_{I_2})(y_{I_1} r_{I_1}^2 - y_{I_2} r_{I_2}^2) \\ &\quad -2(x_{J_1} - x_{J_2})(x_{J_1} r_{J_1}^2 - x_{J_2} r_{J_2}^2) - 2(y_{J_1} - y_{J_2})(y_{J_1} r_{J_1}^2 - y_{J_2} r_{J_2}^2), \\ C &= (x_{I_1} - x_{I_2})^2 + (y_{I_1} - y_{I_2})^2 - (x_{J_1} - x_{J_2})^2 - (y_{J_1} - y_{J_2})^2. \end{aligned}$$

The parameter $k$ can be obtained from the solution of (S7). From the quadratic we have

$$k = \frac{-B \pm \sqrt{B^2 - 4AC}}{2A}. \tag{S8}$$

That is, we get two possible values for $k$. The relevant root of the quadratic is chosen as the one giving the smallest distance after lens distortion correction, where the distance is defined as

$$dis = |(u_{I_1} - u_{I_2})^2 + (v_{I_1} - v_{I_2})^2 - (u_{J_1} - u_{J_2})^2 - (v_{J_1} - v_{J_2})^2|. \tag{S9}$$

## Optimization

In a previous subsection, we described a procedure to estimate the lens distortion parameter $k$ from two points matched from a stereo image pair. It is inevitable, however, that errors are incurred in the process of matching of points in a stereo image pair. As the underlying field is not perfectly flat both fine and coarse scale errors due to the deviation from ideality will naturally arise. In most cases the feature matching process found corresponding points with position errors usually within 1 to 2 pixels. These errors are classified as fine errors and we have used multiple matched points in order to estimate the lens distortion parameters with robustness to fine errors. In a few cases, however, the feature matching process falsely matches points, which can be classified as coarse errors. It is well known that the RANSAC algorithm [8] is robust to coarse errors if these are less than 50%. Therefore, the RANSAC algorithm has been adopted here to avoid the selection of falsely matching points.

Let $N$ denote the number of matched points obtained from the feature matching process. From these we form the $N(N-1)/2$ pairs. The error function utilised in the RANSAC algorithm to evaluate the quality of the estimated parameter is here defined as the sum total of $M$ point pairs with the least errors:

$$\begin{aligned} E_{rr}(k) = \quad & \sum_{i=1}^{M} |[u_{I_{i1}}(k) - u_{I_{i2}}(k)]^2 + [v_{I_{i1}}(k) - v_{I_{i2}}(k)]^2 \\ & -[u_{J_{i1}}(k) - u_{J_{i2}}(k)]^2 - [v_{J_{i1}}(k) - v_{J_{i2}}(k)]^2|. \end{aligned} \tag{S10}$$

where $M < N(N-1)/2$.

The RANSAC algorithm for lens distortion parameter estimation can be summarized as follows.

Step 1 Randomly select a pair of image points;

Step 2 For these, estimate the parameter $k$;

Step 3 Calculate the error using (S10);

Step 4 If the error is within a user-specified tolerance, we accept the estimated parameter k. Otherwise, repeat Steps 1-3;

Step 5 If fail, select the $k$ with the least error.

## References

1. Snavely N, Seitz SM, Szel R. Modeling the world from internet photo collections. International Journal of Computer Vision. 2008;80:189–210.

2. Brown DC. Close-range camera calibration. Photogrammetric Engineering and Remote Sensing. 1971;42:855–866.

3. Rahman T, Krouglicof N. An efficient camera calibration technique offering robustness and accuracy over a wide range of lens distortion. IEEE Transactions on Image Processing. 2012;21(2):626–637.

4. Ahmed M, Farag A. Nonmetric calibration of camera lens distortion: differential methods and robust estimation. IEEE Transactions on Image Processing. 2005 August;14(8):1215–1230.

5. Zhang Z. A flexible new technique for camera calibration. IEEE Transactions on Pattern Analysis and Machine Intelligence. 2000;22(11):1330–1334.

6. Alvarez L, Gomez L, Sendra JR. An algebraic approach to lens distortion by line rectification. Journal of Mathematical Imaging and Vision. 2009;35:36–50.

7. Farid H, Popescu AC. Blind removal of lens distortion. Journal of the Optical Society of America A. 2001;18:2072–2078.

8. Fischler MA, Bolles RC. Random sample consensus: A paradigm for model fitting with applications to image analysis and automated cartography. Communications of the ACM. 1981;24(6):381–395.
